# Anti-Asthma Effect of an Active Components Group from Decoction of *Pheretima Aspergillum* and Its Chemical Composition Characterized by Liquid Chromatography-Quadrupole Time of Flight Mass Spectrometry

**DOI:** 10.22037/ijpr.2019.1100666

**Published:** 2019

**Authors:** Qingxin Shi, Xuanyuan Wang, Junyi Liu, Xingliang Xiang, Mingwu Su, Rongzeng Huang, Chengwu Song

**Affiliations:** *College of Pharmacy, Hubei University of Chinese Medicine, Wuhan, Hubei, China.*

**Keywords:** Asthma, Pheretima aspergillum, Oligopeptides, Active components group, LC-QTOF-MS/MS

## Abstract

The decoction of* Pheretima aspergillum *is used as a traditional medicine for treatment of asthma in China with a long clinical history. The aim of the study was to investigate the anti-asthma activities of an active components group obtained from the decoction of* Pheretima aspergillum *in histamine and ovalubumin induced asthma guinea pigs. The experimental asthma model was established with spray of histamine and ovalbumin (OVA) followed by intragastric administration of *Pheretima aspergillum *extract and fractions. The incubation period of asthma, serum IFN-γ, IL-4, and LTB4 levels were tested and determined. In addition, liquid chromatography-quadrupole time of flight mass spectrometry (LC-QTOF-MS/MS) was used to identify the bioactive components in active fraction. The significant increase of asthma incubation period, serum IFN-γ level were observed in the asthma guinea pigs treated with the active components group. The serum IL-4 and LTB4 levels were obviously decreased compared to that of model controls. In addition, twenty oligopeptides were first identified or characterized in the active fraction from the decoction of* Pheretima aspergillum*. The results indicated that *Pheretima aspergillum* can inhibit infiltration and diffusion of inflammatory cells in asthma model guinea pigs, and regulate IFN-γ, IL-4, and LTB4 secretions. It is reasonable to assume that the anti-asthma activities of *Pheretima aspergillum* mainly resulted from these oligopeptides. Also, synergistic effects among their compounds may exist in the anti-asthma activity of *Pheretima aspergillum*.

## Introduction

Asthma is a common chronic disorder of the airway involving complex interactions among airflow obstruction, airway hyperresponsiveness, and underlying bronchial inflammation (1). The prevalence of asthma has increased in recent decades affecting approximately 334 million people worldwide and ranks third most cause for hospitalization (2). The anti-inflammatory agents were commonly used for treatment of asthma. They can be divided into steroid and non-steroidal agents (3, 4). It is reported that steroidal anti-inflammatory drugs are not universally effective in all patients. Long-term use of those drugs can lead to complications such as pneumonia, fracture, hyperglycemia, and cataract (5). On the other hand, non-steroidal anti-inflammatory drugs (NSAID) are also usually recommended for patients with asthma. NSAID-exacerbated respiratory disease is defined as hypersensitivity to NSAIDs, causing respiratory related symptoms such as bronchospasms, acute asthma exacerbation (lower airway), and severe asthma morbidity (6). NSAIDs, including aspirin, possibly cause asthma exacerbations, particularly in patients allergic to these drugs (7). Because of little adverse effect compared to those of anti-inflammatory agents or other synthetic drugs to prevent or treat such repetitious chronic disease, there are increasing demands for the use of traditional natural drugs in the therapy of asthma (8).

China has 56 ethnic groups that all possess considerable knowledge about the local fauna and flora, exhibiting an array of natural resource use strategies and each ethnic culture possesses a broad knowledge regarding the medicinal properties of wildlife species. While the use of floristic resources in traditional medicine has been widely researched, there is a paucity of information regarding the utilization of faunistic resources. The annelids of Geosaurus (*Pheretima aspergillum *(E. Perrier), *Pheretima vulgaris* Chen, *Pheretima guillelmi* (Michaelsen) and *Pheretima pectinifera* Michaelsen) are valued faunistic Chinese medicines which are listed in the Pharmacopoeia of the People’s Republic of China (2015, edition) for anti-asthmatic remedies (9). Geosaurus preparations, especially their aqueous extracts, have been used widely for medical purposes in China for thousands of years. It have shown to exert biological activities and potential health benefit effects such as invigorating the circulation of blood, stasis-dissolving, antipyretic and diuretic effects (10). The decoction of geosaurus is commonly used in clinical treatment of asthma and possess smooth wheezing step-down, antipyretic analgesic, anticonvulsants, and other unknown pharmacological effects (8). So far, a lot of active ingredients, including amino acids, fatty acids, microelements, lumbritin, lumbrofebrin, terrestrolum brolysin, purine, choline, cholesterin, and vitamins are identified in geosaurus extract (11). However, little information is available regarding to the active components attributed to the anti-asthma effect of geosaurus. 

In this study, seven fractions were obtained by using silica gel column chromatography from total water extract of *Pheretima aspergillum*. Then, the anti-asthma effects were tested among the fractions on asthma of guinea pigs. Meanwhile, the influence of serum leukotriene B4 (LTB4), interleukin 4 (IL-4), interferon-γ (IFN-γ) were determined and the chemical compositions of the active fraction were identified or characterized using liquid chromatography-quadrupole time of flight mass spectrometry (LC-QTOF-MS/MS).

## Experimental

*Chemicals, materials, animals and reagents*

*Pheretima aspergillum *was purchased from Hubei QingDa Chinese Herbal Pieces Co., Ltd. (Shiyan, China). Methanol, ethyl acetate (EtOAc), and cyclohexane were of AR-grade and purchased from Tianjin HengXing Chemical Reagent Co., Ltd. (Tianjin, China). HPLC grade acetonitrile and methanol were obtained from Fisher Scientific (Fair Lawn, NJ, USA). Aminophylline were obtained from Shanghai Yuanye Bio-Technology Co., Ltd (Shanghai, China). Histamine was obtained from Meilun Bio-Technology Co., Ltd, (Dalian, China) and ovalbumin (OVA) was obtained from Sigma Chemical Co. (USA).

Female guinea pigs (250-300 g) were obtained from the Wuhan institute of biological products co., Ltd. (Hubei, China). The animals were kept under standard laboratory conditions of 12-h light-dark cycle and 18-22 °C ambient temperature.

The ELISA kits of IFN-γ, IL-4 and LTB4 were purchased from Wuhan Myhalic Biotechnology Co., Ltd (Wuhan, China).

*Preparation of decoction from Pheretima aspergillum*

After removing the silt, five kilograms of *Pheretima aspergillum* followed by cutting into small pieces (approximately 1 cm × 1 cm). The pieces were decocted in 50 L of distilled water for 3 h, and the extraction process was repeated for twice. The extracts were filtered and concentrated on a rotary evaporator under reduced pressure followed by drying in a freeze-dryer. The extract was subject to silica gel chromatography eluted with different proportions of solvents according to the ascending polarity of cyclohexane-EtOAc and methanol-EtOAc system. As a result, seven fractions were obtained (fraction A-G) with 240 g (4.8%), 265 g (5.3%), 295 g (5.9%), 660 g (13.2%), 835 g (16.7%), 1575 g (31.5%), and 645 g (12.9%), respectively, of the material of *Pheretima aspergillum* (w/w).

*Animal experiments*

The present study was approved by the regional ethics committee for animal research and conforms to the Guide for the Care and Use of Laboratory Animals published by the US National Institute of Health (NIH publication No. 85-23, revised in 1985). In this study, two asthma models were established by histamine and OVA in guinea pigs. First, conscious guinea pigs were placed in a 4L inhalation chamber equipped with a jet nebulizer. The nebulizer was connected to the inhalation chamber air entry, and 20 g L^-1^ histamine solution was delivered for a period of 15 s. The asthma inducing period was counted by a trained observer, and animals that recorded time ranges greater than 120 s were screened through the next test (12). Guinea pigs selected for the study were randomly divided into 11 groups, i.e. normal control group (NC), model control group (MC), positive group (P), water extract group (WE), and fraction A to G groups (F_A_-F_G_), with 8 guinea pigs in each group. The medicines were administered by stomach perfusion once a day for 3 days except guinea pigs in the normal control group after asthma induced by spraying. Aminophylline (0.125 g kg^-1^) was administered to the guinea pigs of positive group; the guinea pigs of MC group received the same volume of physiological saline; the guinea pigs of treatment groups were administrated *Pheretima aspergillum* decoction or fractions, equivalent to 50 g material of *Pheretima aspergillum*.

One hour after the last administration, guinea pigs, except those in the normal control group, were placed in a 4 L inhalation chamber equipped with a jet nebulizer. 20 g L^-1^ histamine solution was delivered for a period of 15 s. The asthma inducing period was counted by a trained observer.

Second, the asthma mode was induced in guinea pigs by inhaled OVA according to the previous literature with minor modifications(13) . Each guinea pig, except those in the NC group, was sensitized by an intraperitoneal injection of 1 mL OVA-physiological saline solution (1:10, w/v) on 0 day. All guinea pigs, except those in the normal control group, were placed in to a 4L inhalation chamber equipped with a jet nebulizer and subject to a 10% (w/v) OVA solution for 30 s on 14th day. The asthma inducing period was counted. The medication was given to the mice on days 15-17. The protocol of the dose described in 2.3.1. One hour after the last administration, the guinea pigs in eight groups were challenged by 10% (w/v) OVA for 30 s. The asthma inducing period was counted.

*Serum IFN-γ, IL-4 and LTB4 levels*

One hour after the last administration, the guinea pigs were anesthetized with intraperitaneous injection of pentobarbital 0.03 g kg^-1^, and the carotid artery was immediately separated to take 5 ml blood. The blood samples were centrifuged for 10 minutes at 3000 rpm. The serum was obtained and stored at -80 °C until use. Serum IFN-γ, IL-4 and LTB4 levels were determined according to the directions of the ELISA kits using an enzyme labeling analyzer (Labsystems Multiskan MS, Finland).

*LC-QTOF-MS/MS analysis*

The fraction C was thawed and dissolved in 50% aqueous methanol (0.5 mg mL^-1^). LC-QTOF-MS/MS was employed to investigate chemical composition of fraction (14). The UHPLC system consisted of a LC-30AD solvent delivery system, a SIL-30AC autosamplerz, a CTO-30A column oven, a DGU-30A3 degasser, and a CBM-30A controller from Shimadzu (Kyoto, Japan). An Ultimate XB-C18 Chromatographic column (3 μm, 3.0 × 150 mm) was used at a flow rate of 0.4 mL min^-1^. The injection volume was 2.0 μL. The column oven was maintained at 30 °C. Mobile phase A was water-formic acid (1000: 1, v/v) and mobile phase B was acetonitrile. The following binary gradient with linear interpolation was used: 0.01 min, 5 % B; 20 min, 43 % B; 27 min, 98 % B; 32 min, 98 % B; 33 min, 5 % B; 35 min, 5 % B.

The QTOF-MS/MS analysis was carried out using a Triple TOF 5600 system with a duo spray source in the positive ion mode (AB SCIEX, Foster City, CA, USA). The QTOF-MS parameters were optimized as follows: ion source temperature, 600 °C; ion spray voltage, 5000 V; curtain gas, 45 psi; nebulizer gas (GS 1), 50 psi; heater gas (GS 2), 50 psi; declustering potential (DP), 80 V. The collision energy (CE) was set at 10 eV for TOF MS/MS. The mass ranges were set at *m/z* 100-1500 for the full-scan mode. The most intensive of 8 ions from each full MS scan were selected as precursor ions for MS/MS fragmentation in the information dependent acquisition (IDA) experiments. Dynamic background subtraction was used to match the IDA criteria. 

*Statistical Analysis*

Experimental values are presented as mean ± SD. Statistical significance was determined by Student′s *t*-test using the SPSS (19.0). Values of *p < 0.05* were considered statistically significant. The acquisition and analysis of LC-MS data were controlled by PeakView^®^ 1.2 software (AB SCIEX, Foster City, CA, USA).

## Results and Discussion

*The incubation period of asthma*

The guinea pigs used in the present study were in good health throughout the experiment and no side effects were observed. The two models used in this study have been widely used for the evaluation of anti-asthma activity with incubation period of asthma as index (13). Table 1 showed that there were no significant differences among the incubation period of asthma before treatment of each group (*p > 0.05*). In addition, the marked decrease in incubation period of asthma in MC group indicated a successful establishment of an asthma model. As depicted in Table 1, the incubation period after treatment in positive group significantly increased as compared with those in the MC group (*p < 0.01*). Meanwhile, Fraction C possessed potent anti-asthma effect by the evidence of significantly increased asthma incubation period when compared to that of other treatment groups.

*Serum IFN-γ, IL-4 and LTB4 levels*

As shown in Table 2, the serum IFN-γ, IL-4, and LTB4 levels in NC group were within the normal ranges in two models. In the asthma guinea pigs induced by histamine, significantly decreased serum IFN-γ levels were observed in guinea pigs of MC group, compared to that of guinea pigs in NC group. Significantly increased serum IL-4 and LTB4 levels were observed in guinea pigs of MC group, compared to that of guinea pigs in NC group. A successful establishment of an asthma model was demonstrated. There were significant reductions in the serum IFN-γ value in P and F_C_ groups, which were 78% and 76%, respectively, higher than that of MC group. The serum IL-4 levels in the P and F_C_ groups, represented a significant increase of 59% and 54% as compared to that in MC group, respectively. The serum LTB4 levels in the P and F_C_ groups in the asthma guinea pigs induced by histamine, represented an increase of 67% and 74% as compared to that in MC group, respectively. In addition, the serum IFN-γ, IL-4 and LTB4 levels showed the same trends in the asthma guinea pigs induced by OVA.

**Table 1:** The incubation period of asthma induced by histamine and OVA in guinea pig (mean ± *SD*, n = 8).

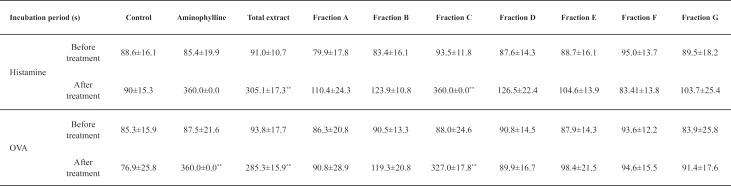

* *p < 0.05*, ** *p < 0.01 *significantly different from control groups.

**Table 2:** The serum IFN-γ, IL-4 and LTB4 levels in asthma guinea pig induced by histamine and OVA (mean ± *SD*, n = 8).

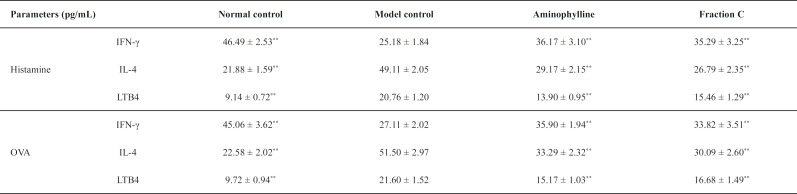

* *p < 0.05*, ** *p < 0.01 *significantly different from model control groups.

**Table 3:** LC-QTOF-MS/MS identification of oligopeptides in fraction C obtained from the decoction of *Pheretima aspergillum*.

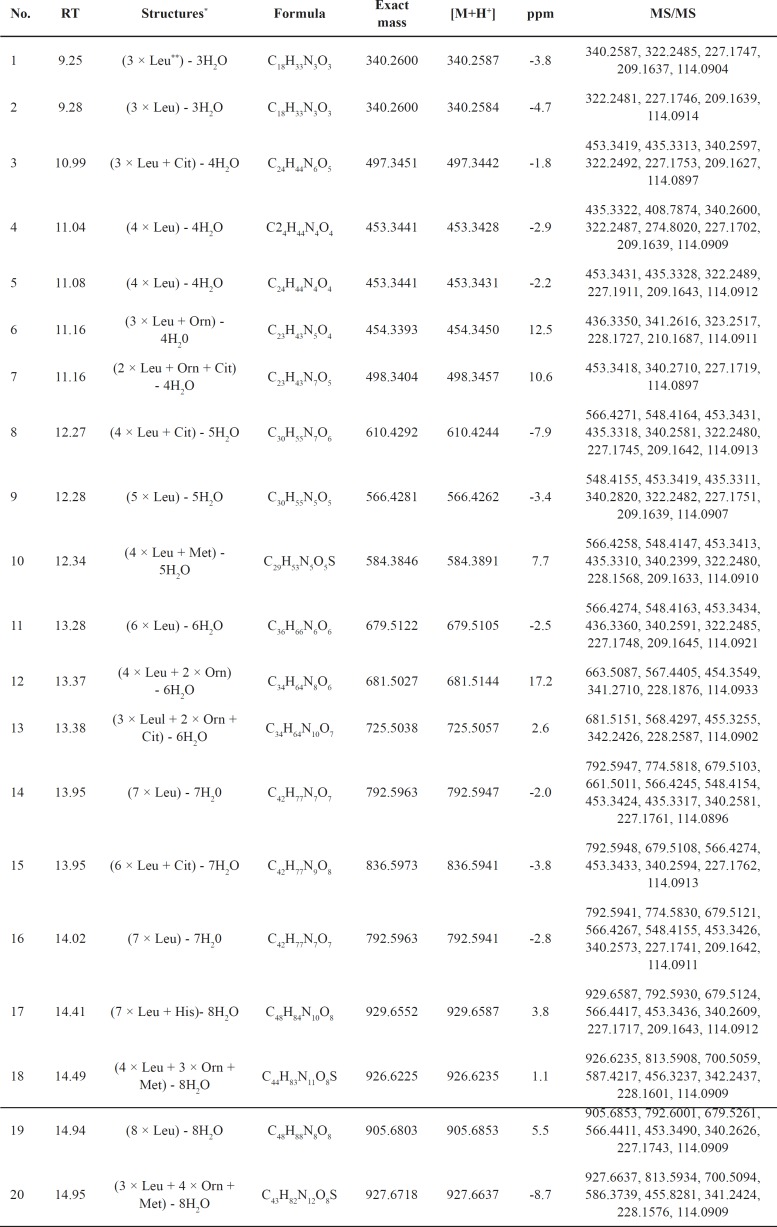

* Abbreviations: Leucine (Leu); Isoleucine (Ile); Ornithine (Orn); Methionine (Met); Histidine (His); Citrulline (Cit).

** Within the limitations of LC-MS/MS, the isomeric amino acids, such as Leucine and Isoleucine could not be determined by LC-QTOF-MS/MS in this study.

**Figure 1:**
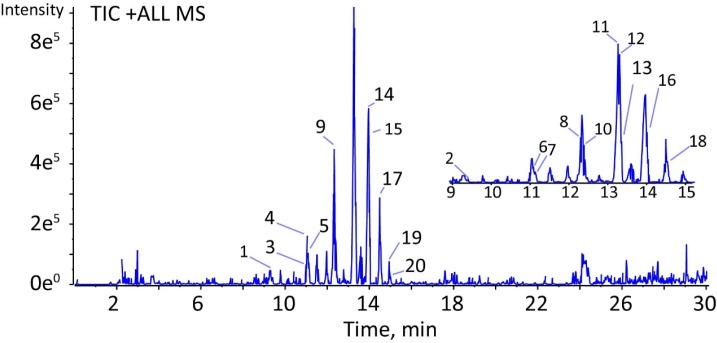
TIC mass spectrum in positive mode of fraction C by LC-QTOF-MS/MS.

As the best species of Pheretima in traditional Chinese medicine, numerous Chinese medical scholars have recognized *Pheretima aspergillum* as an effective medicine for various activities (15-17). The recent study reported that decoction of *Pheretima aspergillum* effectively mitigated Th2 type inflammatory reactions (5, 18). It significantly decreased the levels of IL-4, IL-5 and IL-13 and downregulated IgE expression (19). In addition, the decoction of *Pheretima aspergillum* has an attenuated effect on mucus secretion and infiltration of inflammatory cells in the lung (20). T-lymphocyte subgroups and the levels of Th1/Th2 cytokines played an important role in the occurrence of bronchial asthma, and severity of patient′s condition and subsequent therapeutic effects (21). The cytokines released by the T-lymphocyte subgroups exert a critical role on the inflammatory reactions of the respiratory mucosa and mycoplasma pneumonia. The main function of Th1 cells is to secrete IFN-γ, which can induce the activation of macrophages and generate immunoglobulin, mainly characterized by cytotoxic effects (22). During the induction of secretion of macrophages and mast cells, the important function of Th2 cells is to secrete IL-4, which are mainly characterized by delayed type hypersensitivity (23). Infiltration of inflammatory cells into airway tissues was associated with expressions of specific adhesion molecules enhanced by Th2-mediated cytokines such as IL-4 (24). In the present study, the increaesd IFN-γ and decreased IL-4 levels in serum gives evidence for the ameliorative effect of fraction C in treatment of asthma.

In the recent experiment, it was found that *Pheretima aspergillum* can decrease the serum LTB4 level in the asthma guinea pigs (20). In addition, the decoction of *Pheretima aspergillum* not only influences the inflammatory cells, such as eosinophil and lymphocytes, but also participates in the immune regulation to regulate the cytokines, such as LTB4 (17). Leukotrienes are a group of proinflammatory lipid mediators produced by many different cells of the innate immune system, including granulocytes, mast cells, and macrophages (25). In asthma specifically, LTB4 was found to be elevated in bronchoalveolar lavage, plasma, and sputum (26). Meanwhile, LTB4 is related to asthma exacerbations and the development of airway hyperresponsiveness (27). In the present study, the ameliorative effect of fraction C on the levels of serum LTB4 levels was observed, indicating that the anti-asthma activity of *Pheretima aspergillum *may be possibly attributed to the regulations of proinflammatory lipid mediators. 

*LC-QTOF-MS/MS analysis*

The characterization of the chemical composition in fraction C was completed mainly by rationalization of their data of accurate mass measurement and the collision-induced dissociation (CID) MS/MS product ions. As depicted in Figure 1, twenty compounds were characterized and extracted in a total ion chromatogram (TIC). All of the precursor ions exhibited a very similar product ion at m/z 114.0919, suggested the same substituent on the structures of these compounds. First, the neutral loss of monosaccharide substituents was excluded from the analyses. Second, a series of amino acids were examined to fit the regular fragmentation patterns of these compounds. For instance, compound 9 showed a predominant precursor ion at m/z 566.4267 (calculated for C_30_H_55_N_5_O_5_, 566.4281) in positive ion mode. As is shown in Table 3, the product ion at m/z 113.0841 was easily produced by cleavage of the peptide bond. Since the product ions at m/z 114.0919 is a characteristic indicator for the presence of leucine. The compound 9 was characterized as an oligopeptide composed of 5 leucines. Comparing with the structure information of 24 amino acids, the other compounds were characterized based on the same procedures above. As a result, twenty oligopeptides were characterized in fraction C (Table 3). However, within the limitations of LC-MS/MS, the isomeric amino acids, such as leucine and isoleucine could not be determined by LC-QTOF-MS/MS in this study.

The organic solvents are commonly used in the extraction methods for pharmacology research. However, those methods could not reflect the real characteristic active components of some Traditional Chinese Medicine material because most of them were consumed in the form of boiling water extraction, which would result in different phytochemical profiles in the extracts. Amino acids are one of the major components of *Pheretima aspergillum*, which have been studied as active components that possessed anti-asthma effects (28). Oligopeptide, being a major intracellular redox regulator has been shown to be implicated in regulation of airway reactivity and inflammation. Severe asthmatics had biochemical changes related to steroid and amino acid/protein metabolism (29). The depletion or repletion of glutathione exacerbates or ameliorates allergic asthma respectively by regulation of airway oxidante antioxidant balance (30). The complex oligopeptides characterized in fraction C obtained from the decoction of* Pheretima aspergillum* suggested that the amino acids were complex during boiling water extraction process. Therefore, the oligopeptides identified in the fraction C may in part contributed to the anti-asthmatic effect of *Pheretima aspergillum*.

## Conclusion

In this study, the anti-asthma effect of an active components group from decoction of *Pheretima aspergillum* were investigated using two asthma models *in-vivo*. The components group effectively increased the incubation period of asthma induced by histamine and OVA in guinea pig. Moreover, twenty oligopeptides were first identified or characterized by LC-QTOF-MS/MS in fraction C from the decoction of *Pheretima aspergillum*. Our results provide evidence that the oligopeptides may have potential anti-asthma effect in the development of bronchial asthma. However, due to the complexity of the chemical composition in *Pheretima aspergillum*, the exact bioactive compound(s) that is/are responsible for the anti-inflammatory and anti-asthmatic effects or the synergistic effects among these compounds are still unknown and should be further investigated in our future studies.
